# Metastatic Clear Cell Renal Cell Carcinoma Masquerading as Urinary Bladder Tumor: A Report of a Rare Case

**DOI:** 10.7759/cureus.105364

**Published:** 2026-03-17

**Authors:** Shreyash Garg, Ankur Mittal, Vikas K Panwar, Deelip Kumar Singh, Ashish V Tekwani

**Affiliations:** 1 Urology, All India Institute of Medical Sciences, Rishikesh, Rishikesh, IND

**Keywords:** bladder metastasis, clear cell renal cell carcinoma, immunohistochemistry, sunitinib, transurethral resection of bladder tumor

## Abstract

Clear cell renal cell carcinoma (ccRCC) is known to be the most common histological subtype of renal cell carcinoma, with a well-known potential for distant metastasis. Common metastatic sites are the lungs, liver, bones, and brain; however, metastasis to the urinary bladder is exceptionally rare. We describe a 64-year-old man with a history of high-grade ccRCC who developed painless gross hematuria six months after undergoing right radical nephrectomy. Cystoscopic evaluation revealed two polypoidal bladder masses. Histopathological and immunohistochemical analyses confirmed metastatic ccRCC (positive for carbonic anhydrase IX (CAIX), paired-box gene 8 (PAX8), and cluster of differentiation 10 (CD10)). The patient was managed by transurethral resection of bladder tumor (TURBT) followed by targeted therapy with sunitinib (37.5 mg). He remains disease-free at one-year follow-up. This case underscores the importance of considering metastatic recurrence in patients with a prior history of RCC who present with new-onset urinary tract symptoms. Although rare, bladder metastasis should be included in the differential diagnosis. The underlying mechanism remains speculative, with possible routes including hematogenous spread, retrograde venous dissemination, or direct seeding. ccRCC can metastasize to unusual sites, including the urinary bladder. Early recognition through comprehensive imaging and immunohistochemical evaluation is essential for timely diagnosis and management.

## Introduction

Renal cell carcinoma represents nearly 2%-3% of all malignancies in adults, of which the clear cell variant accounts for approximately 70%-80% of cases [1]. Metastatic dissemination is a well-recognized feature of advanced RCC, most commonly involving the lungs, liver, bone, and brain. Metastasis to the urinary bladder is exceedingly uncommon, with only isolated cases reported in the literature [2,3].

This report describes a rare case of metachronous bladder metastasis developing six months after nephrectomy for primary clear cell renal cell carcinoma (ccRCC). This report emphasizes the importance of maintaining a high index of suspicion and coordinated multidisciplinary care in cases with atypical recurrence.

## Case presentation

**Figure 1:**
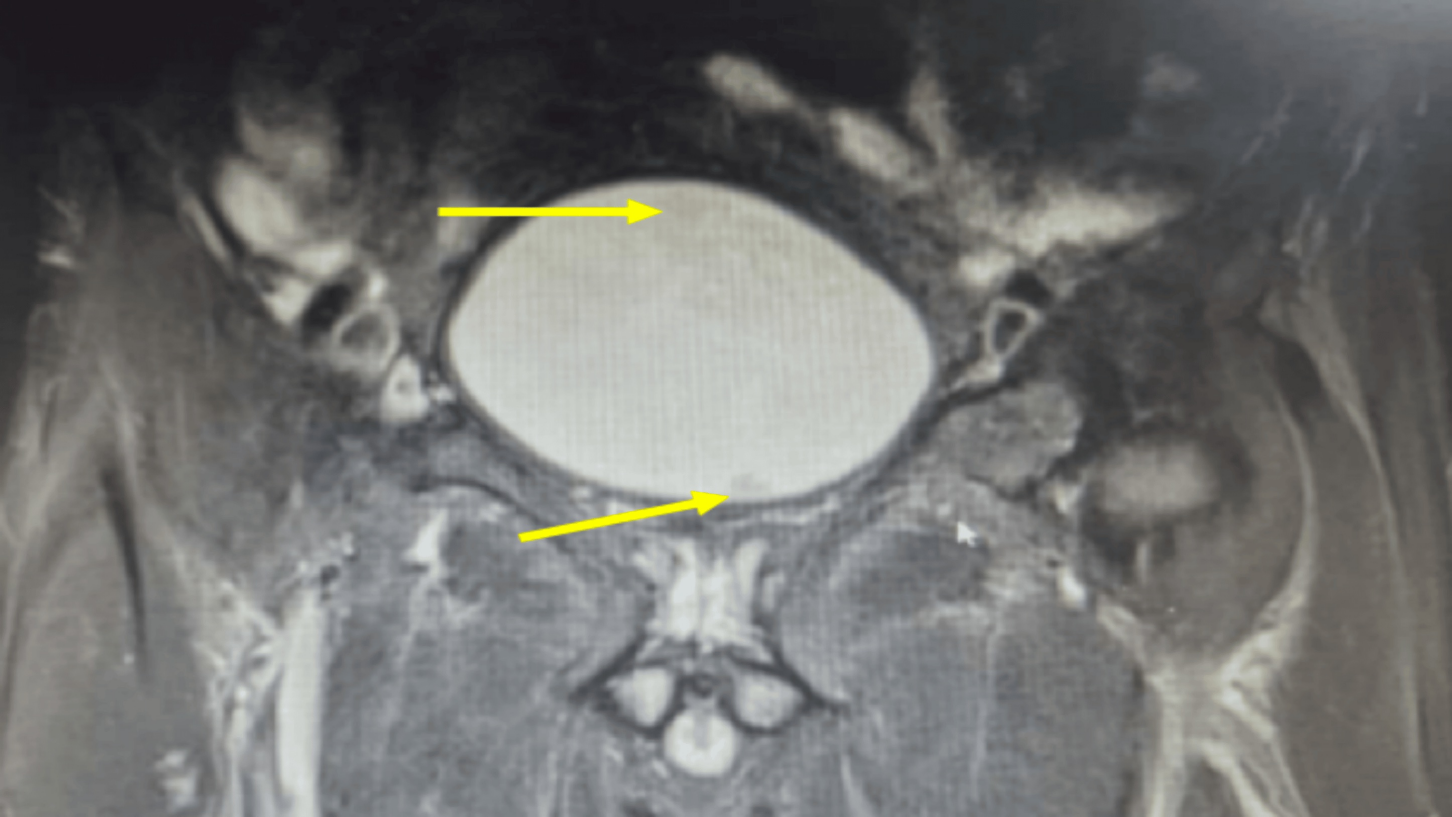
MRI of the pelvis showing polypoidal lesions at the bladder dome and base T2-weighted MRI showing a 3.5 cm lesion arising from the bladder dome (arrow) and a 1.6 cm lesion arising from the bladder base (arrow) MRI: magnetic resonance imaging

**Figure 2:**
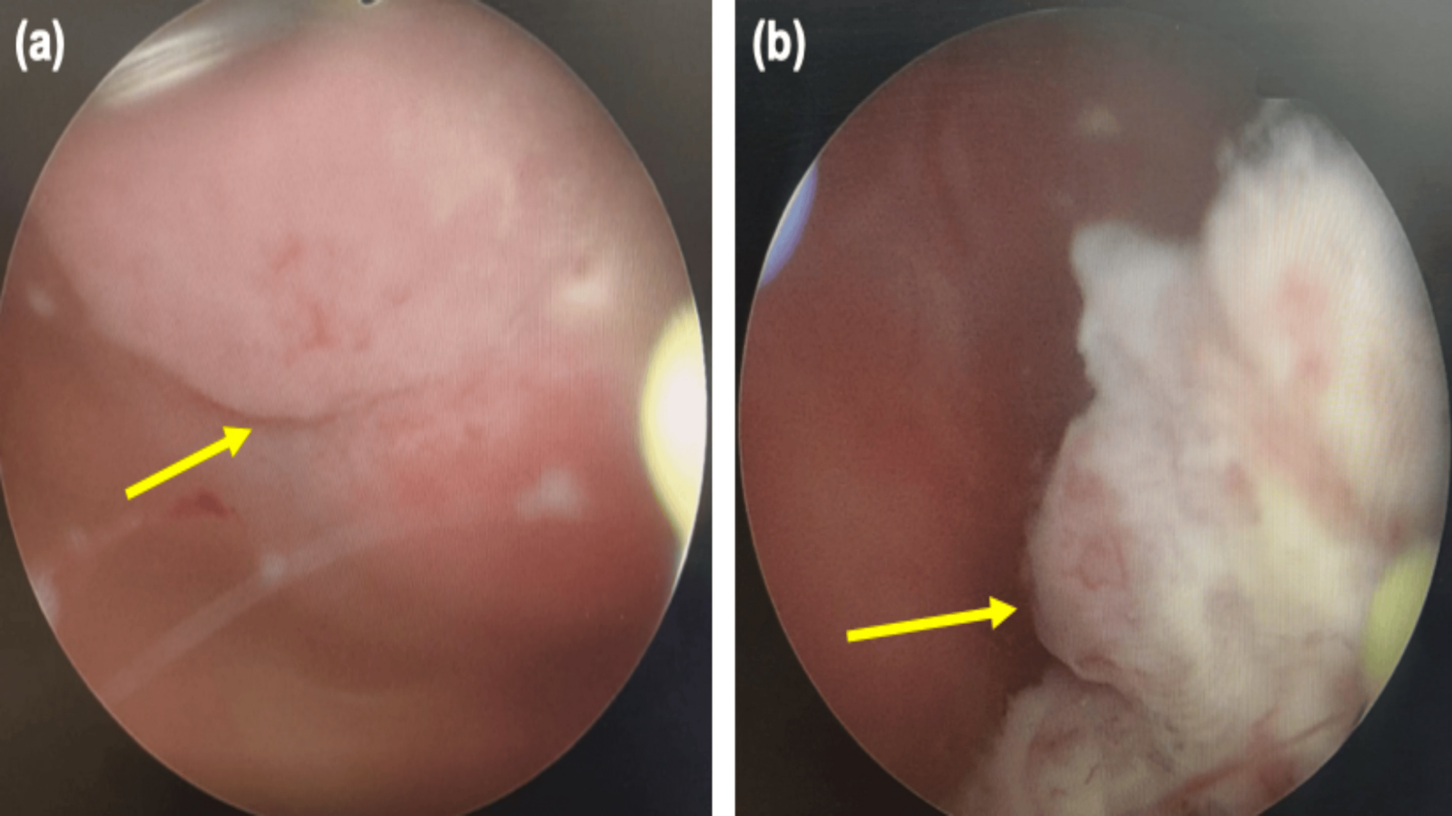
Cystoscopic appearance of metachronous bladder metastasis from clear cell renal cell carcinoma (a) Large, polypoidal, solid bladder lesion with smooth surface and broad base seen at the dome (arrow). (b) Smaller, well-defined polypoidal lesion visualized at the bladder base (arrow).

A 64-year-old man with an Eastern Cooperative Oncology Group (ECOG) performance status of 1 and no significant comorbidities presented with gross painless hematuria and passage of clots six months after undergoing right radical nephrectomy for high-grade clear cell renal cell carcinoma with upper ureteral involvement (pT3aN0M0). Magnetic resonance imaging (MRI) performed 13 months after right radical nephrectomy demonstrated two well-defined polypoidal lesions in the urinary bladder, one located at the dome measuring 3.5 cm and another at the base measuring 1.6 cm (Figure 1). Subsequent cystoscopic examination confirmed the presence of two solid polypoidal bladder lesions corresponding to the dome and base locations (Figures 2a, 2b). The patient underwent transurethral resection of bladder tumor (TURBT), and complete endoscopic resection of both lesions was achieved.

**Figure 3:**
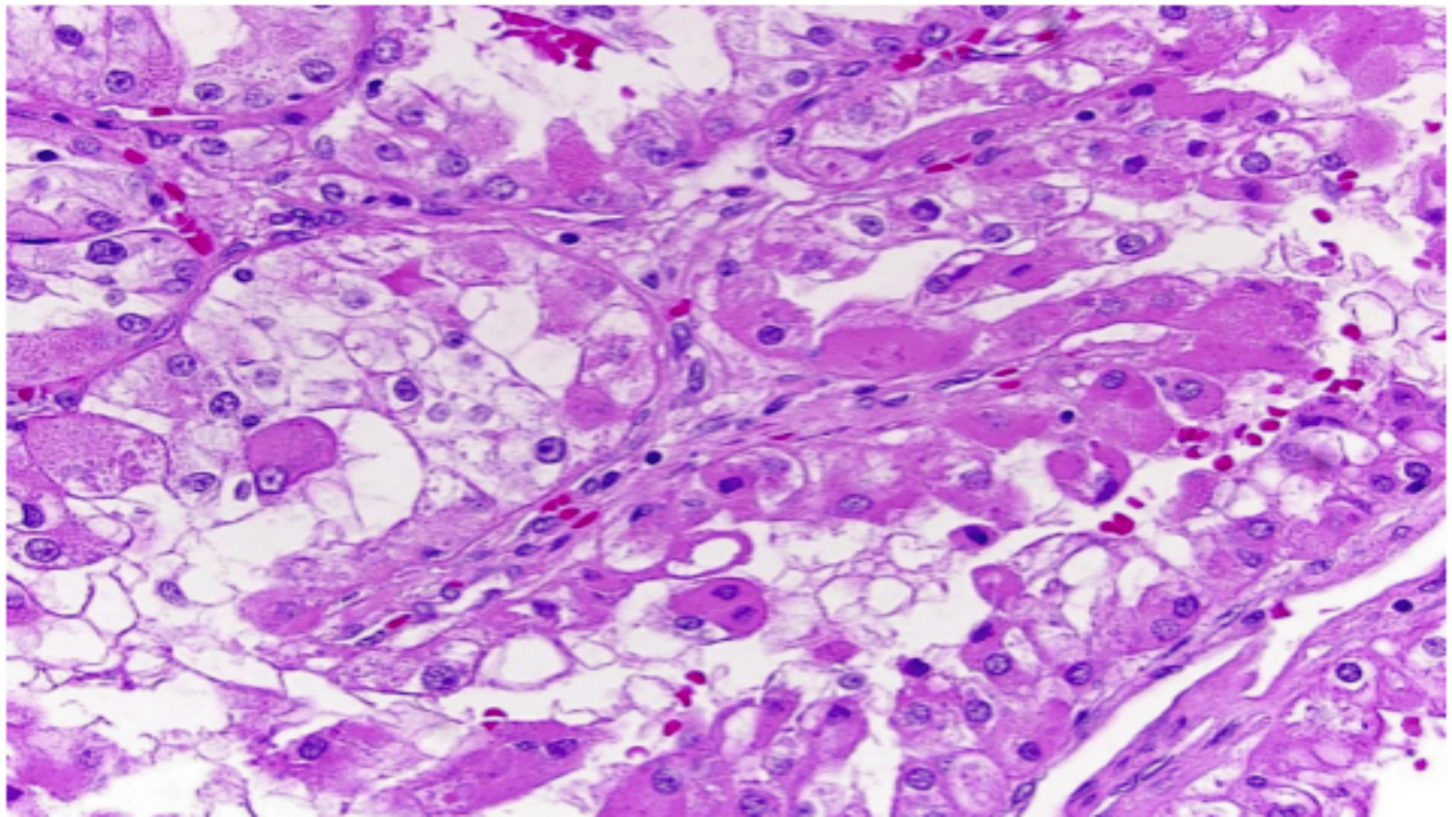
Histopathology of the bladder lesion (H&E, 20×) Tumor cells with clear cytoplasm arranged in a nested pattern consistent with metastatic RCC H&E: hematoxylin and eosin, RCC: renal cell carcinoma

**Figure 4:**
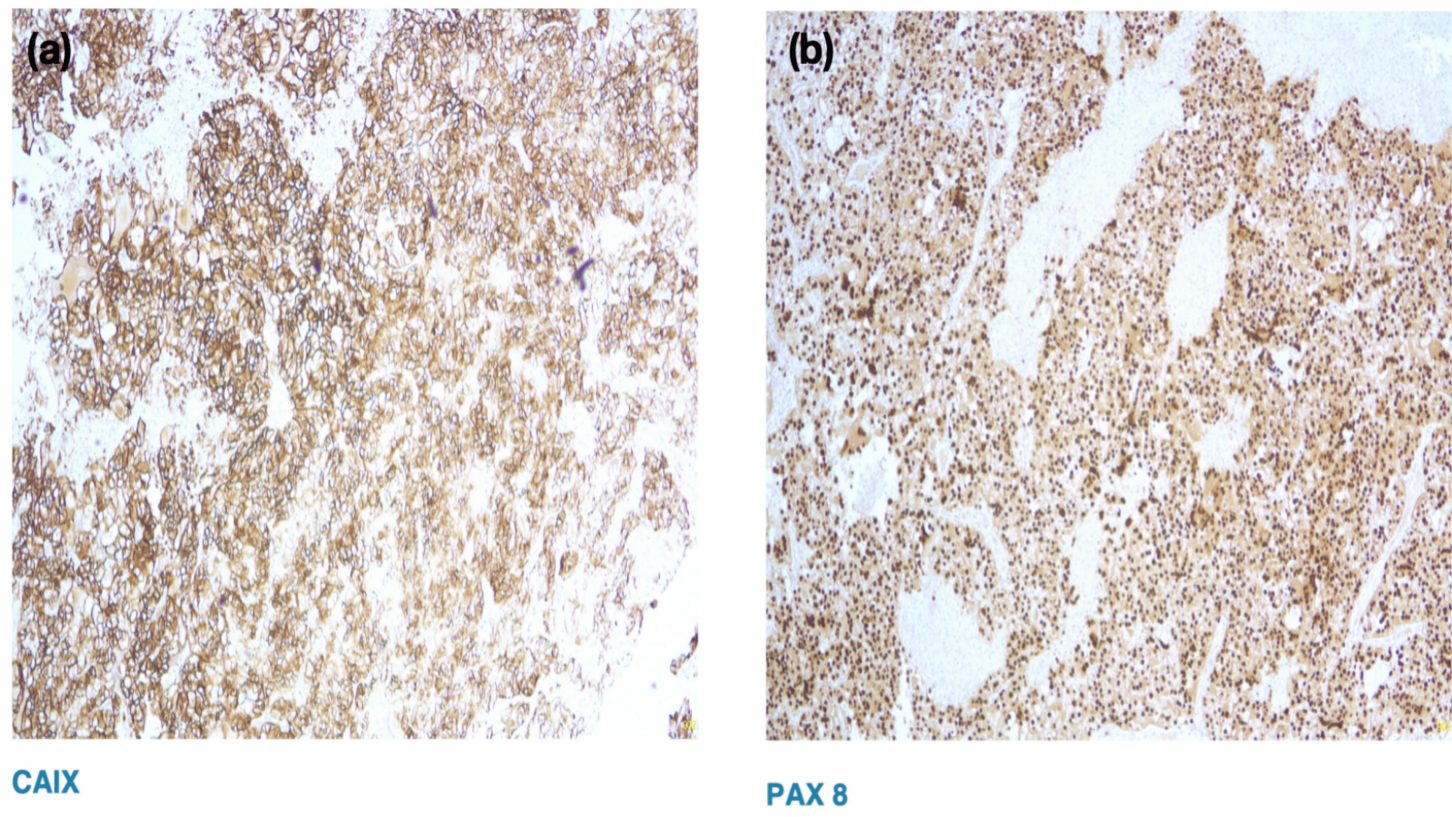
Immunohistochemistry showing CAIX and PAX8 positivity (a) Tumor cells showing CAIX positivity. (b) Tumor cells showing PAX8 positivity. CAIX: carbonic anhydrase IX, PAX8: paired-box gene 8

Histopathological examination revealed tumor cells with clear to eosinophilic cytoplasm arranged in nested and alveolar architectural patterns, consistent with metastatic clear cell renal cell carcinoma (Figure 3). Immunohistochemical analysis showed strong positivity for carbonic anhydrase IX (CAIX) with characteristic box-like membranous staining, along with positivity for paired-box gene 8 (PAX8), confirming the renal origin of the tumor (Figures 4a, 4b).

Following histological confirmation, the patient was started on sunitinib 37.5 mg once daily as maintenance targeted therapy. At 12-month follow-up after resection, the patient remained asymptomatic, with no evidence of recurrence on cystoscopic evaluation and radiological surveillance.

## Discussion

Metastasis of RCC to the urinary bladder is an exceptionally rare event, with fewer than 100 cases documented globally [2,3]. Bladder involvement is reported in only 0.3%-1.6% of RCC cases, highlighting the unusual nature of this metastatic pattern [4,5]. Bladder metastases from RCC may present either synchronously or metachronously [4,5]. Most reported cases, approximately 77%, are metachronous, occurring after treatment of the primary renal tumor. The median interval between nephrectomy and detection of bladder metastasis is around 33 months. Notably, recurrence within the first 12 months following nephrectomy has been associated with poorer cancer-specific survival, suggesting more aggressive tumor biology in early relapses [5].

The precise mechanism by which ccRCC metastasizes to the bladder remains incompletely understood. Several pathways have been proposed. Hematogenous dissemination through systemic circulation or via renal vein involvement is one plausible explanation. Retrograde venous or lymphatic spread through periureteral or pelvic channels has also been described [4]. Another widely discussed hypothesis is intraluminal tumor seeding, or “drop metastasis,” in which malignant cells travel along the urinary tract and implant in the bladder mucosa. This mechanism is particularly suspected when the primary tumor involves the pelvicalyceal system or collecting ducts [4,5]. Furthermore, the presence of hematuria at the time of primary RCC diagnosis, even in the absence of a detectable bladder lesion, has been suggested as indirect evidence of tumor cell shedding through the urinary apparatus [5].

On histopathological examination, metastatic ccRCC to the bladder has features similar to ccRCC, typically demonstrating nests of polygonal cells with abundant clear cytoplasm separated by a delicate and rich vascular network. Immunohistochemistry is essential for confirming renal origin and excluding primary clear cell tumors of the bladder. Markers such as PAX8 and CAIX support a diagnosis of metastatic RCC. Conversely, negative staining for CK7, CK20, CDX-2, UROPLAKIN III, and GATA3 helps rule out primary urothelial carcinoma and clear cell adenocarcinoma of the bladder. Immunohistochemistry plays a pivotal role in differentiating metastatic RCC from primary urothelial carcinoma, CAIX and CD10 positivity being highly indicative of renal origin [6]. Accurate differentiation is critical, as management strategies differ significantly between metastatic RCC and primary bladder malignancies, where bladder metastases from RCC require immunotherapy/tyrosine kinase inhibitors in addition to TURBT in comparison to primary bladder malignancies requiring TURBT alone [4].

Prognostically, isolated bladder metastasis, when amenable to complete resection, is associated with favorable outcomes compared to widespread metastatic disease [2].

This case aligns with previously reported literature, reinforcing the notion that bladder metastasis may occur even in patients with apparently localized RCC [2,3]. Early diagnosis through vigilant follow-up and targeted therapy can achieve durable remission.

## Conclusions

Bladder metastasis from ccRCC is a rare but clinically significant entity. Awareness among clinicians and urologists is essential for prompt recognition, especially in patients presenting with new urinary tract symptoms post-nephrectomy. Multimodal management, encompassing endoscopic resection, histopathological confirmation, and systemic therapy, remains the cornerstone for optimizing patient outcomes.
